# Insulin oxidation and oxidative modifications alter glucose uptake, cell metabolism, and inflammatory secretion profiles

**DOI:** 10.1016/j.redox.2024.103372

**Published:** 2024-10-05

**Authors:** Ramona Clemen, Wiebke Dethloff, Julia Berner, Paul Schulan, Alice Martinet, Klaus Dieter Weltmann, Thomas von Woedtke, Tilman Grune, Kristian Wende, Sander Bekeschus

**Affiliations:** a ZIK Plasmatis, Leibniz Institute for Plasma Science and Technology (INP), 17489, Greifswald, Germany; b Department of Dermatology and Venerology, Rostock University Medical Center, 18057, Rostock, Germany; c Institute for Hygiene and Environmental Medicine, Greifswald University Medical Center, Ferdinand-Sauerbruch-Str., 17475, Greifswald, Germany; d German Center for Cardiovascular Research (DZHK), Partner Site Berlin, 10785, Berlin, Germany; e German Center for Diabetes Research (DZD), 85764, Muenchen-Neuherberg, Germany; f Department of Molecular Toxicology, German Institute of Human Nutrition Potsdam-Rehbruecke (DIfE), Nuthetal, 14558, Germany; g Department of Physiological Chemistry, Faculty of Chemistry, University of Vienna, Vienna, 1090, Austria

**Keywords:** Cold physical plasma, CAP, Gas plasma technology, kINPen, Mass spectrometry, Reactive oxygen species, ROS, OxPTM

## Abstract

Insulin participates in glucose homeostasis in the body and regulates glucose, protein, and lipid metabolism. Chronic hyperglycemia triggers oxidative stress and the generation of reactive oxygen species (ROS), leading to oxidized insulin variants. Oxidative protein modifications can cause functional changes or altered immunogenicity as known from the context of autoimmune disorders. However, studies on the biological function of native and oxidized insulin on glucose homeostasis and cellular function are lacking. Native insulin showed heterogenous effects on metabolic activity, proliferation, glucose carrier transporter (GLUT) 4, and insulin receptor (INSR) expression, as well as glucose uptake in cell lines of five different human tissues. Diverse ROS compositions produced by different gas plasma approaches enabled the investigations of variously modified insulin (oxIns) with individual oxidative post-translational modification (oxPTM) patterns as identified using high-resolution mass spectrometric analysis. Specific oxIns variants promoted cellular metabolism and proliferation in several cell lines investigated, and nitrogen plasma emission lines could be linked to insulin nitration and elevated glucose uptake. In addition, insulin oxidation modified blood glucose levels in the chicken embryos (in ovo), underlining the importance of assessing protein oxidation and function in health and disease.

## 1 Introduction

Increased blood sugar levels are regulated by pancreatic β cell-secreted insulin and amylin. The vital polypeptide hormone insulin enables the activation of a series of kinase cascades in body cells by binding to its receptor (INS1R), which triggers various cell responses and has been summarized elsewhere [[1], [2], [3]]. In brief, insulin increases the permeability of the cell membrane for glucose uptake by the glucose carrier transporter (GLUT4), for example, in skeletal muscle and adipocytes. Consequently, insulin regulates cell growth and proliferation by activating the transcription of genes. These metabolic or mitogenic effects of insulin are initiated in target tissues such as liver [4], muscle [5], and fat [6,7], but also cancer cells [[8], [9], [10]].

However, the failure of the INS1R, referred to as insulin resistance, leads to increased insulin secretion (hyperinsulinemia) and failed blood sugar level regulation, resulting in severe symptoms of hyperglycemia. These diseases, which indicate Diabetes mellitus type I and II, are characterized by an altered metabolism of glucose [11], fat, and proteins and are one of the most serious and common chronic diseases of current times [12]. Dysregulated glucose homeostasis triggers premature senescence of various cell types, including pancreatic response (reviewed in Ref. [13]). Further hallmarks of Diabetes include increased insulin resistance due to chronic hyperglycemia leading to glucolipotoxicity, oxidative cell stress, and inflammation [14,15]. Indeed, a pathophysiological finding in type 1 diabetes (T1D) is chronic inflammation and research has started to focus on low-grade inflammation (LGI) in type 2 diabetes (T2D), accompanying the development and progression of the disease. Inflammation and oxidative cell stress correlate with increased levels of reactive oxygen and reactive nitrogen species (RONS) [[16], [17], [18]], including superoxide (O_2_^•-^), hydroxyl radical (HO^•^), peroxynitrous acid/peroxynitrite (ONOOH/ONOO^−^), hydrogen peroxide (H_2_O_2_) [19,20], and hypochlorite Ions forming hypochlorous acid (HOCl) [[21], [22], [23], [24]]. Those RONS can cause antioxidant deficiency [25], as well as severe damage to cellular macromolecules as lipid peroxidation [26,27], protein alterations in structure and function due to oxidative post-translational modifications (oxPTMs), and DNA strand breaks by radical attack.

Multiple studies have previously provided evidence that hyperglycemia-mediated generation of oxidants triggers oxidative stress in T1D and T2D [[28], [29], [30], [31]]. Consequently, oxPTMs occur [32], and antibodies targeting oxidized insulin have been identified in patients' blood samples [32,33]. Additionally, there is evidence that oxPTM on insulin-derived epitopes, commonly associated with T1D, improves T-cell binding capacity and may be responsible for activation in T1D and subsequent β-cell death [33].

However, studies on the biological function of oxidized insulin on glucose homeostasis are lacking, although an environment rich in H_2_O_2_ promotes glucose uptake and AKT phosphorylation [34]. Taking into account that different RONS oxidize insulin to different extents and lead to various effects, we used physical cold plasma technology (also called, e.g., cold atmospheric plasma, low-temperature plasma, and gas plasma) [[35], [36], [37]] with varying gases in our study, thereby creating a modular multi-ROS environment. This allowed us to identify the oxPTMs on insulin-induced by different ROS using high-resolution mass spectrometry. We here investigate the effects of oxidized insulin on metabolic activity, proliferation, GLUT4, and INSR expression, as well as glucose uptake in cell lines of five different human tissues, namely lung carcinoma cells A549, non-tumorigenic hepatoma HepG2 cells, adenocarcinoma MCF7 cells, lymphoblast TK6 cells, and leukemia monocytic cells THP-1. We further investigate glucose uptake after incubation with oxidized insulin in rat pancreatic INS1E cells, human-INSR-expressing Chinese hamster ovary cells CHO-INS1R, and testes glucose regulation in chicken embryos (*in ovo*) after injecting native or oxidized insulin.

## 2 Results

### 2.1 Insulin response differed in cells originating from different tissues, and metabolic activity was dose-dependent

**Fig. 1:**
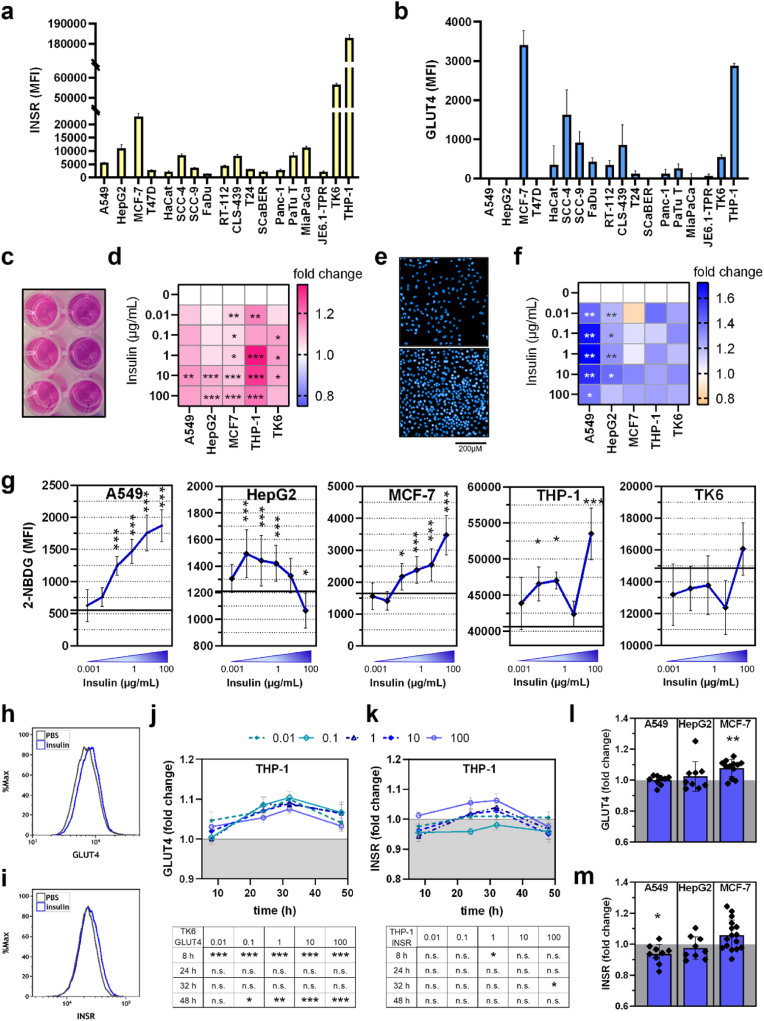
**Native insulin promotes metabolic activity in five different cell lines and affects proliferation and surface marker expression.** Expression of surface markers (**a**) insulin receptor (INSR) and (**b**) glucose transporter (GLUT4) was determined in cells of different origin by flow cytometry; five cell lines were selected for further studies and incubated with different insulin concentrations before (**c, d**) metabolic activity and (**e,f**) proliferation were measured; (**g**) glucose uptake was determined by adding a fluorescent glucose probe (2-NBDG). Data are shown as mean ± SEM from three to five independent experiments with three to six technical replicates for each measurement. Statistical analysis was performed by the one-way analysis of variance (ANOVA), comparing insulin-treated cells vs. vehicle-treated cells (∗ = p < 0.05, ∗∗ = p < 0.01, ∗∗∗ = p < 0.001, n.s. = not significant); (**h**) representative histogram of GLUT4 signal and (**j, l**) quantified signals; (**i,k,m**) INSR signal in different cell lines after adding native insulin. **d, f, j-m** are mean values that include data from three to five independent experiments with three to six technical replicates for each measurement. Statistical analysis was performed by the Mann-Whitney test, comparing insulin-treated cells vs. vehicle-treated cells (∗ = p < 0.05, ∗∗ = p < 0.01, ∗∗∗ = p < 0.001, n.s. = not significant). **j, k** are mean values ± SEM from three biological replicates, including three technical replicates for each measurement. Statistical analysis was performed by the two-way analysis of variance (ANOVA), comparing treated samples versus untreated proteins for each time point (∗ = p < 0.05, ∗∗ = p < 0.01, ∗∗∗ = p < 0.001, n.s. = not significant).

To evaluate which cell line is suitable for an insulin response based on its receptor occurrence, we first screened 18 cell lines of our stock for the surface marker expression of insulin receptor (INSR) and glucose receptor (GLUT4) by flow cytometry (Fig. 1a and b). Interestingly, some of the cell lines that had a higher INSR expression also showed high GLUT4 expression (e.g., THP-1 and MCF-7), and some of the low INSR expressing cells showed the lowest expression of GLUT4 (e.g., A549, T47D, SCaBER, and JE6.1-TPR). Leukemia cells THP-1, TK6, and breast cancer cells MCF-7 showed the highest INSR expression, but THP-1, MCF7, and squamous cell carcinoma cell SCC4 showed the highest GLUT4 expression. Other cells that expressed an average number of INSR had almost no GLUT4 expression (e.g., HepG2 and MiaPaCa). For further analysis, we chose ordinary cell lines having different tissue origins and representing a group of varying expression levels: adenocarcinoma MCF-7 cells for high receptors expression, lung carcinoma cells A549 for low INSR levels, and non-tumorigenic hepatoma HepG2 cells for average INSR expression. Furthermore, high INSR lymphoblast TK6 cells and leukemia monocytic cells THP-1 were investigated. Cells were incubated with different concentrations of native (untreated, in PBS) human insulin, and the metabolic activity was determined after adding resazurin, which is converted to fluorescent resorufin by cell metabolism-derived NADPH in a 1:1 stoichiometry (Fig. 1c and d). MCF-7 and THP-1 significantly increased metabolic activity after adding 0.01 μg/ml insulin, while other cell lines needed higher concentrations. After incubation with 10 μg/ml, all cell lines showed raised metabolic activity. However, comparing metabolic activity to proliferative capacity, A549 and HepG2 divided after adding insulin most, but not MCF7, THP-1, or TK6 (Fig. 1e,f, and S1a). Stimulation with ten μg/mL also led to cytokine secretion, which was further investigated after 48h by multiplex analysis (Fig. S1b). Herein, different cell lines showed individual cytokines up or downregulated compared to vehicle (PBS). Immune cells THP-1 and TK6 cells mainly decreased the secretion of all investigated cytokines or showed no difference, whereas A549 and MCF-7 showed increased levels of pro-inflammatory cytokines, e.g., monocyte chemoattractant Protein-1 (MCP1), interleukin (IL) 6, IL8, IL12, and IL18.

### 2.2 Insulin increased glucose uptake and modestly altered surface marker expression of INS1R and GLUT4

We next evaluated glucose uptake using fluorescent glucose 2-NBDG after adding insulin at different concentrations, and dose-dependent effects were determined. Increasing amounts of insulin correlated with increasing glucose uptake in A549, THP-1, and MCF-7, but decreasing 2-NBDG signals were measured in HepG2 cells (Fig. 1g). Important to mention that in immune cell lines, THP-1 and TK-1, the baseline level is ten times higher than in non-immune cells. Glucose uptake is initiated by increasing the number of membrane-bound GLUT4 receptors after INSR is internalized and the signaling pathway activated. Consequently, we next evaluated the surface marker expression of GLUT4 and INSR after incubation with insulin via flow cytometry (Fig. 1h and i). In THP-1 cells, 8h after adding insulin, GLUT4 surface expression started to increase with having a maximum after 32h and significantly elevated levels compared to control cells (Fig. 1j). Albeit INSR1 expression was slightly reduced after incubation with 1 μg/ml insulin, higher concentration (100 μg/ml) led to raised levels in THP-1 cells (Fig. 1k). TK-6 and MCF-7 cells increased GLUT4 expression after 8h incubation, too, but not A549 (Fig. 1k–S1c), while INS1R was reduced in TK-6 and A549, but not in MCF-7 (Fig. 1l–S1c). Aiming to investigate the effect of oxidized insulin on cells, we next treated insulin with different ROS profiles by physical gas plasma technology. We analyzed the cellular response to oxidized insulin in the other cell lines.

### 2.3 Gas plasma treatment structurally altered insulin

**Fig. 2:**
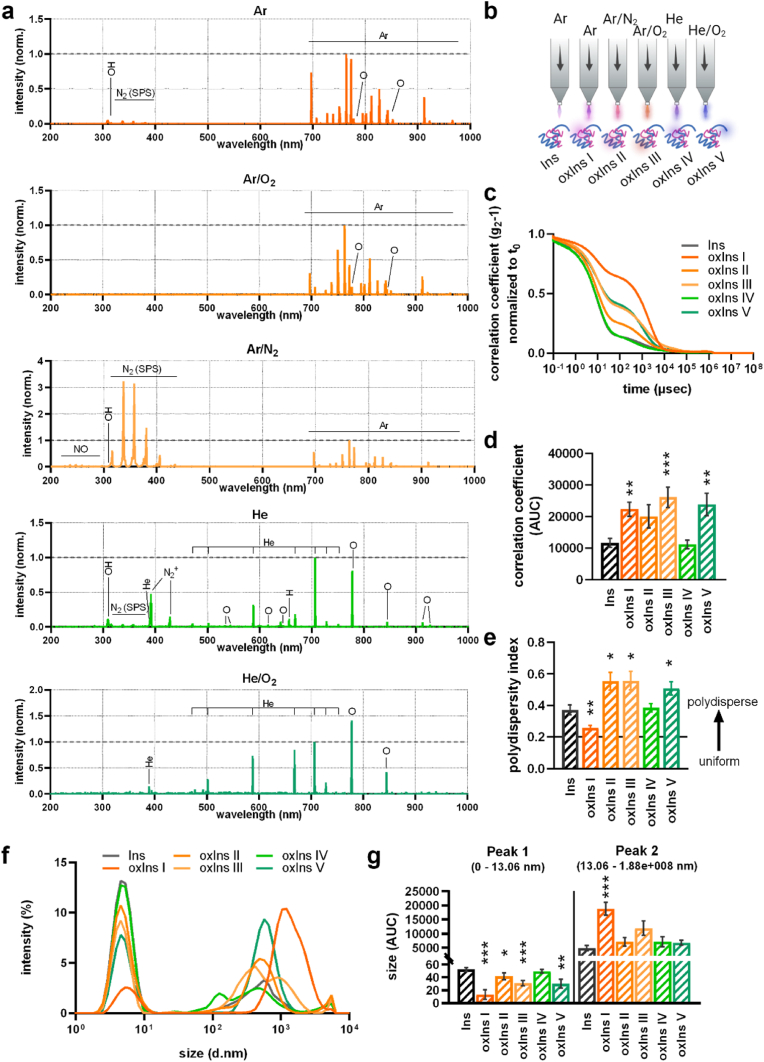
**Cold physical plasma generated reactive oxygen species that affect insulin's structure.** (**a**) Reactive species generated by cold physical plasma varied when using different feed gases to ignite the plasma; (**b**) scheme of insulin exposed to argon gas as control or five different plasma conditions; effects on insulin were determined by dynamic light scattering, where (**c, d**) correlation coefficient, (**e**) polydispersity index, and (**f, g**) size were determined. **d, e, g** are mean values ± SEM from six independent experiments with three technical replicates each. Statistical analysis was performed by the Mann-Whitney test, comparing treated samples versus untreated proteins (∗ = p < 0.05, ∗∗ = p < 0.01, ∗∗∗ = p < 0.001, n.s. = not significant).

**Fig. 3:**
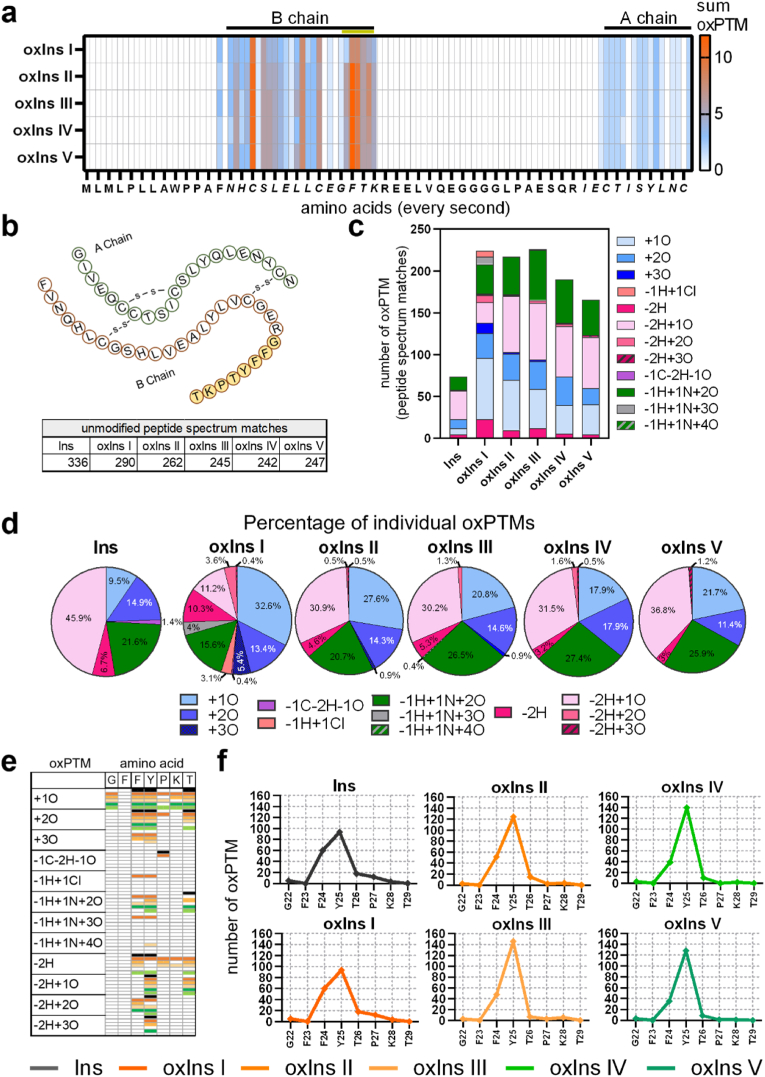
**Identification of oxidative post-translational modifications in oxidized insulin.** Insulin was exposed to different plasmas, and oxPTMs were identified by mass spectrometry analysis. (**a**) overview of summarized oxPTM distribution of the A and B chain sequences (the B chain sequence GFFYTPKT with high PSM numbers marked in yellow); (**b**) A and B chain's amino acid sequence and PSM count of unmodified GFFYTPKT sequence; (**c**) total number of oxPTMs detected in native insulin and plasma-treated insulin variants; (**d**) percentage of individual oxPTMs in oxidized insulin variants; (**e**) individual oxPTMs on specific amino acids in the detected (GFFYTPKT) sequence; (**f**) accumulated oxPTMs on amino acids of the GFFYTPKT sequence. (For interpretation of the references to colour in this figure legend, the reader is referred to the Web version of this article.)

Suggesting that oxidative stress and chronic inflammation-generated ROS affect insulin's function, we determined size, correlation coefficient, and polydispersity via circular dichroism spectroscopy after plasma treatment. Cold physical plasma is known for generating a plethora of short-lived and long-lived reactive species [38], and using different feed gas varies the generated ROS [39]. Here, five plasmas were used by ionizing the feed gases argon (Ar), argon-oxygen (Ar/O_2_), argon-nitrogen (Ar/N_2_), helium (He), or helium-oxygen (He/O_2_) before optical emission spectroscopy was applied to identify reactive species in the plasma (Fig. 2a). A line corresponding to hydroxyl radicals (OH), was found in Ar, Ar/N_2_, and He, but not in Ar/O_2_ and He/O_2_ plasma. Furthermore, nitrogen (N_2_) was generated in Ar, Ar/O_2_, Ar/N_2_, and He plasma, and atomic oxygen was determined in Ar, Ar/O_2_, He, and He/O_2_. Next, 1 mg/ml insulin was exposed to different plasma conditions to generate oxIns I, II, III, IV, and V (Fig. 2b). For control experiments, insulin was exposed to argon gas only, without ionization (Ins). The correlation coefficient, which indicates the degree of non-randomness, changed after treatment with four of five plasma conditions, with three showing significant changes (Fig. 2c and d). However, helium plasma did not lead to alteration of the correlation coefficient of insulin (oxIns IV), which was in line with no changes in the polydispersity index (Fig. 2e). In contrast, oxIns I showed a more uniform dispersity, while oxIns II, oxIns III, and oxIns V showed a significant increase in polydispersity. Increased polydispersity also correlated with a shift of size in different populations (Fig. 2f and g). Indeed, with the exception of oxIns IV, all oxidized variants showed reduced signals at a size from 0 to 13.06 d nm, which corresponds to molecules with molecular weights smaller than 100 kDa. As a dominant structure, larger particles were determined in all conditions, and the highest signal was found in oxIns I, suggesting an aggregation process. However, Coomassie and silver staining only revealed signals for monomeric structure, and no oligomers or aggregates were determined (Figs. S2a–c), at least for the lithium dodecyl sulfate highly reducing buffer used with heating at 70 °C for 10 min. Assuming that reactive species not only affect the protein's structure but also induce oxidative modifications on amino acids, we further performed mass spectrometry to identify oxPTMs in insulin after plasma treatment (see Fig. 3).

### 2.4 Gas plasma exposure oxidized A and B chains of insulin

Before oxPTMs on insulin peptide's amino acids were examined after exposure to different plasmas via nano LC-MS, we performed a control experiment to assess the reproducibility of our method to introduce, measure, and analysis oxidative modifications on peptides using gas plasma technology. In an experimental series of five independent sample preparations of the artificial peptide SIINFEKL, the number of modifications identified did not only significantly differ from that of argon gas-treated peptides but also had an overall modest fluctuation (fig. S3a). Compared to the control peptide, showing already about one-third of peptides containing at least one modification (Fig. S3b), the gas plasma-treated peptides were majorly modified (about two-thirds) and showed a much greater variety of oxidative modifications (fig. S3c). Regarding insulin, we identified several amino acids of the A chain and B chain of the mature insulin (Fig. 3a), and more oxPTM were identified in the B chain sequence compared to the A chain. However, only one part of the B chain (23–29 GFFYTPK) gave high peptide spectrum matches (PSM) and was suitable for further analysis (marked in yellow, Fig. 3a,b). Interestingly, untreated insulin showed 336 PSM of unmodified GFFYTPK_23-29_, whereas 242 to 290 unmodified PSMs were identified in insulin after treatment with different plasma conditions (Fig. 3b). Furthermore, two to three times more oxPTM were detected in the oxIns variants, compared to untreated in insulin (Fig. 3c). Argon plasmas induced similar total numbers of oxPTM in oxIns I, II and III (all above 200), oxIns IV showed total oxPTMs of 180 and oxIns V only 160. A more detailed view of the oxPTMs revealed similar modifications with different distributions (Fig. 3d). Indeed, oxidation (+1O), dioxidation (+2O), nitration (-1H+1 N+2O), and carbonylation (-2H+1O) were the most abundant oxPTMs in untreated, as well as in oxidized variants (Fig. 3e). However, the percentage of accumulated individual oxPTMs among all oxPTMs identified differed between the groups (i.e., not the absolute number of oxPTMs but the relative importance of each oxPTM among all oxPTMs identified when analyzed globally and not on a per-amino-acid-resolution). For instance, in native (untreated) insulin, 9.5 % of all types of oxPTMs identified by mass spectrometry were of the type "oxidation", indicating already at baseline a presence of such modification due to, e.g., sample preparation and oxygen or hydrolysis-derived ROS. In plasma-treated insulin variants, in contrast, the portion of oxidation among all types of modifications analyzed was at least 15 % (32.6 % in oxIns I, 27.6 % in oxIns II, 20.8 % in oxIns III, 17.9 % in oxIns IV, 21.7 % in oxIns V). Dioxidation was found to a similar extent in all samples, and carbonylation was reduced in plasma-treated insulin variants. Trioxidation (+3O) was specifically found in argon plasma-treated insulin (I, II, and III), and quinone (-2H+2O) was detected in oxIns I, II, III, and IV. Importantly, the heterogenic effects were found on the amino acid level in native insulin and oxidized variants (Fig. 3e). For instance, trioxidation in oxIns I and II similarly occurred on F24 and Y25. Still, trioxidation in oxIns III was only determined on Y25. Nitration on Y25 only appeared in oxIns variants but not in native insulin. We did not identify oxPTM on F23, which may result from the algorithm-based analysis that cannot distinguish occurring oxPTM on F at positions 23 and 24. Interestingly, oxPTM on G22 and K27 were only detected in plasma-oxidized insulin variants but not in native proteins. The highest number of oxPTMs (in relative terms to other oxPTMs) was found on tyrosine (Y25) (in a majority but not all peptides measured) in all conditions investigated (Fig. 3f). To investigate further cellular effects of the oxPTMs, we analyzed several aspects in cells after incubation of plasma-treated insulin.

### 2.5 Gas plasma-oxidized insulin increased cellular metabolic activity

**Fig. 4:**
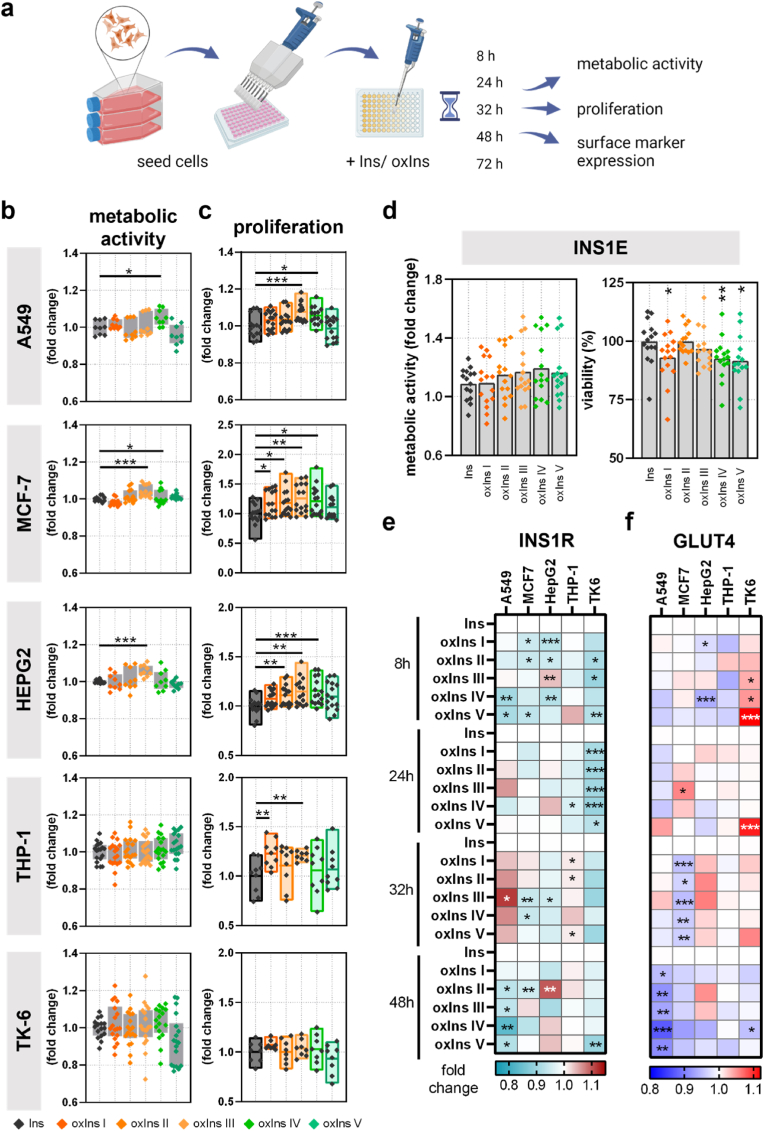
**Oxidized insulin promotes metabolic activity, proliferation, and INSR and GLUT4 expression.** (**a**) workflow of the study in which different cell lines were seeded and incubated with native or oxidized insulin for various durations before different aspects were determined; (**b**) metabolic activity was determined 8h after adding insulin or oxidized insulin by resazurin assay; (**c**) proliferation was determined by high-content imaging and flow cytometry; (**d**) metabolic activity and viability in pancreatic beta cells after incubation with native or oxidized insulin; after the cells were incubated with Ins or oxIns variants, surface marker expression of (**e**) INSR and (**f**) GLUT4 was determined by flow cytometry. **b-f** are mean values ± SEM from three to six independent experiments with three technical replicates each. Statistical analysis was performed by the Mann-Whitney test, comparing treated samples versus untreated proteins (∗ = p < 0.05, ∗∗ = p < 0.01, ∗∗∗ = p < 0.001, n.s. = not significant). **e,f** are mean values of three biological replicates with three technical replicates each. Statistical analysis was performed by the two-way analysis of variance (ANOVA), comparing treated samples versus untreated proteins for each time point (∗ = p < 0.05, ∗∗ = p < 0.01, ∗∗∗ = p < 0.001, n.s. = not significant).

Suggesting that oxPTMs alter the biological effect of insulin, we investigated metabolic activity, proliferation, glucose uptake, and INSR and GLUT4 expression after incubation with the different oxInsulin variants (Fig. 4a). Interestingly, each cell line showed individual effects on metabolism after different durations in response to oxidized insulin. Many of the cell lines increased their metabolic activity in the presence of some oxIns variants, and none of the Plasma conditions led to a significant reduction of the metabolism (Fig. 4b). This was only observed after 8h, but also after 24h, 32h, 48h and 72h (Fig. S4a). OxIns I did not affect metabolic activity after 8h in any of the cell lines but showed increased levels at later times in TK-6 cells (32h), MCF-7, and HEPG6 (both 72h). A similar was observed after incubating cells with oxIns II, which raised the activity in TK-6 (24h) and MCF-7 cells (72h). OxIns III led to increased metabolism in MCF-7 and HEPG2 (8h and 72h) and A549 (32h). OxIns IV showed raised metabolism in A549 (8h and 32h) in MCF-7 (8h and 72h), and oxIns V only affected the metabolic activity of MCF-7 after 72h incubation. Oxidized insulin did not affect THP-1 cell metabolism, but oxIns I and oxIns III promoted cell proliferation and led to increased cell number (Fig. 4c). Cell proliferation was also increased in MCF-7, HEPG2, and A549, but not TK-6. Considering the fact that insulin is naturally produced in pancreatic beta cells and oxidative stress occurs in Diabetes that may affect insulin, we further investigated the effects of oxidized insulin on INS1E cells. Albeit the metabolic activity was not affected after incubation with oxIns, we observed small changes in cell viability (Fig. 4d). Indeed, oxIns I, oxIns IV, and oxIns V slightly reduced the number of viable cells by 5–7%. However, we did not focus further on the small changes in pancreatic cells but rather on the receptor equipment on the cells, as these are relevant for the interaction with insulin.

### 2.6 Altered surface marker expression and cytokine secretion with oxidized insulin

Native protein altered metabolic activity in a dose-dependent manner without correlation with GLUT4 and INS1R expression or with an overall response observed in all cell lines. Similar unconditional changes in GLUT4 and INS1R surface marker expression after 8h incubation with oxidized insulin were determined in the different cell lines independent of metabolic activity and proliferation (fig. Sb). Confirming the expectation that glucose uptake is initiated by reduced INSR and increased GLUT4 receptor expression, we also found synergistic effects for some conditions, e.g., in TK-6 cells (Fig. 4e and f). Indeed, TK-6 cells showed decreased INS1R signals and increased GLUT4 expression after 8h, 24h, and 32h incubation with oxidized insulin, and similar but not significant patterns were determined in HEPG2 after 32h (Fig. 4e,f and S5a,b). Interestingly, INS1R reduction mainly correlated with decreased GLUT4 expression, as observed in A549 cells (48h), MCF-7 (32h), HEPG2 (8h), and TK-6 (48h). Elevated INS1R expression was only observed in A549 after 32h when incubated with oxIns, which correlated with increased secretion of monocyte chemoattractant protein-1 (MCP-1), interferon (IFN) α, and interleukin (IL)6, 8, 18 and 23 (Fig. 4e and S6a). MCF-7 showed reduced MCP-1, IL6, and IL8 secretion after incubation with oxIns V (Fig. S6b), whereas HEPG2 did not show notable changes in cytokine secretion after incubation with oxidized insulin (Fig. S6c). Interestingly, in supernatants of oxIns stimulated monocytic THP-1 cells, higher concentrations of the pro-inflammatory cytokine IL6 and anti-inflammatory cytokine IL10 were detected (Fig. S6d). In TK6 cells, oxIns I, II, and III led to reduced levels of most of the determined cytokines, whereas oxIns IV and V promoted the secretion of some cytokines (Fig. S6e).

### 2.7 Oxidized derivates led to reduced glucose uptake in A549, MCF-7, and pancreatic islets but promoted glucose uptake in INS1R-overexpressing cells

**Fig. 5:**
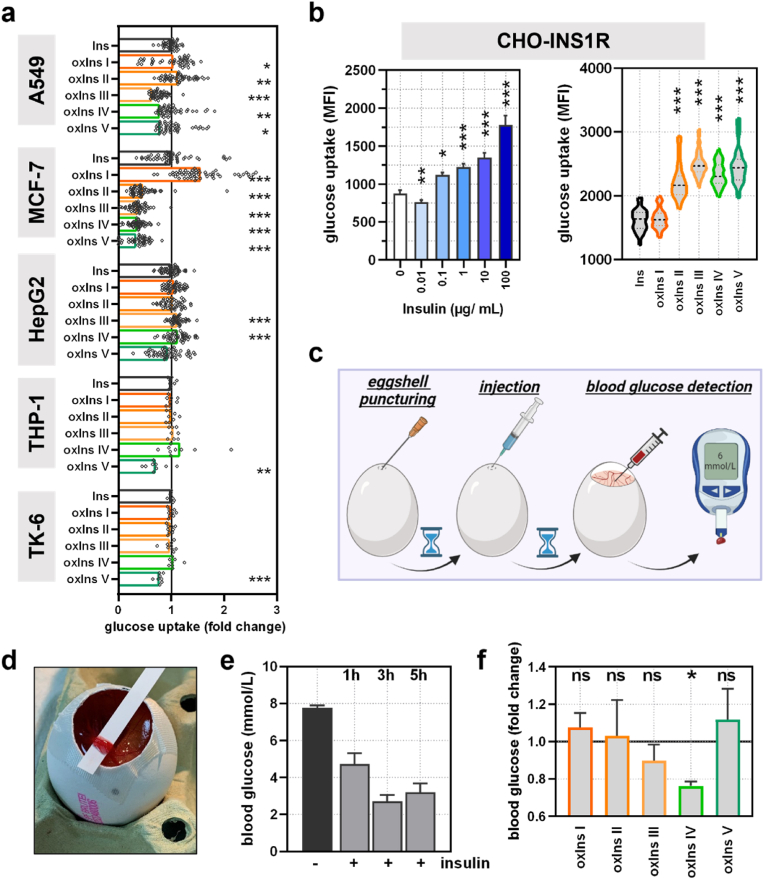
**Glucose uptake is increased after incubation with oxidized insulin *in vitro* but not *in ovo*.** (**a**) glucose uptake was determined by adding a fluorescent glucose probe (2-NBDG) together with native or oxidized insulin and measuring fluorescent intensity after 1 h. Data are shown as mean ± SEM from three independent experiments with three or 12 technical replicates each; (**b**) transgenic, human INSR expressing CHO cells were incubated with Ins or oxIns and 2-NBDG to determine glucose uptake. Data are shown as mean ± SEM from three independent experiments with three or 12 technical replicates each; (**c**) workflow of *in ovo* experiment; (**d**) photo of a blood vessel separated from the chicken egg to take blood for blood glucose testing; (**e**) Ins was applied on the CAM following determination of blood sugar level after one, three and 5 h; (**f**) blood glucose level in eggs 3 h after adding native or oxidized insulin. All individual data points are shown Data are shown as mean ± SEM from five to eight independent experiments. **a-c,e-f** Statistical analysis was performed by the Mann-Whitney test, comparing Ins vs. oxIns (∗ = p < 0.05, ∗∗ = p < 0.01, ∗∗∗ = p < 0.001, n.s. = not significant).

The question that arose was whether oxIns-mediated changes lead to altered glucose uptake. To this end, cells were pre-incubated with native or oxidized insulin, and fluorescently labeled glucose was added to the cells to determine the glucose uptake rate. Interestingly, oxIns I was the one condition that led to increased glucose uptake in A549 and MCF-7 cells. In contrast, most other conditions reduced the glucose uptake rate (Fig. 5a). Indeed, oxIns V significantly reduced the uptake rate in all wild-type cell lines. OxIns III, as well as oxIns IV led to lower signals in two out of five cell lines. Albeit oxIns II led to reduced glucose uptake in MCF-7, this oxidized protein increased the uptake rate in A549 but did not affect the uptake rate in HEPG2, THP-1, or TK-6. The overall response was heterogeneous in terms of cell line and oxidized variant, considering the results of investigated parameters, namely cytokines, metabolic activity, proliferation, surface marker expression, and glucose uptake (Fig. S6f). Along similar lines, an overall correlation between INS1R expression and glucose uptake for the respective cell lines and oxidized variants of insulin was not observed (Fig. S6g). The association of INS1R expression and glucose uptake rate was further tested in genetically modified, INS1R overexpressing Chinese hamster ovary cells (CHO-INS1R). Here, increasing concentration of insulin led to increased glucose uptake, and apart from oxIns I, all conditions elevated the glucose uptake rate (Fig. 5b).

### 2.8 Insulin oxidation affected systemic glucose regulation in ovo

To evaluate in a more complex system whether the oxidized insulin reduces or promotes glucose uptake, we tested glucose reduction in an *in ovo* model using fertilized chicken hen eggs. The eggshells of fertilized chicken hen eggs were punctuated prior to the application of native or oxidized insulin on the chorioallantoic membrane (CAM) and incubated at breeding conditions before blood was taken to determine blood sugar level (Fig. 5c and d). The blood sugar level drastically dropped 3 h after insulin injection (Fig. 5e). Interestingly, oxIns IV improved the effect of native insulin. At the same time, none of the other oxidized variants showed significant effects compared to its native form (Fig. 5f).

## 3 Discussion

Hyperglycemia leads to increased concentration of free radicals and other reactive species [40] that affect biomolecules undergoing oxidative modifications with pathological consequences. In the context of autoimmunity in T1D, oxPTMs and their immunological consequences have been studied extensively; however, cellular response to oxidized insulin regarding INSR and GLUT4 regulation, metabolism, proliferation, and glucose uptake were omitted. In this study, we determined an overall heterogeneous cellular response in terms of cell line and oxidized variant. Cytokine secretion, metabolic activity, proliferation, surface marker expression, and glucose uptake in cell culture experiments were affected by different oxIns variants. Furthermore, most oxIns variants significantly increased glucose uptake in transgenic, INSR-overexpressing CHO cells, albeit only oxIns V showed systemic glucose regulation in chicken eggs. The inconsistent results may be caused by additive effects of other cells that react to oxIns.

In line with previous results, the main response of native insulin in A549 [41], as well as in MCF7 cells [8,10], was stimulating metabolism and cell proliferation, and we observed a similar trend in THP-1 and HepG2 cells. Intriguingly, the effect was dose-depend for some cell lines, as a higher concentration increased metabolic activity, but in HepG2 a higher dose reduced metabolic activity. Further studies with other cell lines will be needed to address the point of heterogenic responses in future work. Regarding oxidized variants, we further observed promoted effects in most of the investigated cell lines after incubation with oxidized insulin. Evidence on the cellular effects of oxidized insulin is rare to find in the literature. Of note, a reduced biological activity of insulin oxidized by a metal-catalyzed oxidation system (H_2_O_2_/Cu) was found in adipose tissue [42]. However, this cell type was not investigated in the present study. Mechanistically, insulin leads to the phosphorylation of Akt (pAkt), which plays a crucial role in the linkage of GLUT4 to the insulin signaling pathway [4,9,43]. We suggest similar effects in the present study as we previously found oxIns I to increase pAkt in primary human monocytes [44]. We further observed heterogenic responses regarding the different oxIns variants that were used, such as oxIns III and oxIns IV were more potent to stimulate proliferation, and oxIns V was unable to affect metabolism or proliferation. One reason can be the individual oxPTM, which differs in plasma-treated insulin, and further when comparing plasma-treated insulin with insulin after treatment with conventional ROS. Albeit the overall PSM number of A chain sequence GIVEQCCTSICSLYQLENYCN was low and therefore excluded in our study for further analysis, previous studies identified oxidized Y14 and C20 after ROS exposure [[45], [46], [47]].

Consistent with the results of these studies, where oxidized amino acids F25 and Y26 in B chain sequence ERGFFYTPKT were found after incubation with alternative ROS, we identified oxidation in the same amino acids after exposure to plasma-generated ROS. Important to mention here that +1O and +2O on F25 and Y26 also occurred on native insulin (probably during the preparation process), but +3O were individual modifications after exposure to argon-plasma I, II, and III. We further identified oxPTM on G22 and K27 in B chain sequence GFFYTPK, which were specifically detected in plasma-oxidized insulin variants but not in native proteins. We suggest that glutamine in insulin is an important scavenger to prevent hyperoxidation, as glutamine has antioxidant capacity in Diabetes, and supplementation reduces oxidative stress [48,49]. Importantly, possible scavenger functions may prevent cysteines from being modified, which are important for the interaction between the A chain and B chain and within the A chain. Cysteine oxidation indicates the cleavage of disulfide bonds, leading to structural changes, as previous studies have reported, due to oxidative damage [50,51]. Insulin exposed to ROS has previously been shown to aggregate by cross-linking at different positions [46], and also fibril formation of insulin has been described [52,53]. In line with the previous results, we also found higher formed oligomers in insulin after treatment with three of five plasma conditions, suggesting altered cross-linking events. Besides intramolecular cross-links, intermolecular cross-linking events occur when insulin binds to its receptor, regulating cellular responses. Previous studies show that residue F24 (B chain) and V3 (A chain) could be cross-linked to the first leucine-rich-repeat domain (L1) of the receptor A chain and that residues Y15 and F23 of B chain insulin could be cross-linked to the N-terminal region of the receptor (reviewed in Refs. [54,55]). In other words, the direct interaction of insulin with the C-terminal region of the insulin receptor is restricted to three B chain residues and one A chain residue in insulin. Intriguingly, we found a high number of oxPTM on F24 and suspect an altered binding to the receptor, explaining the altered cellular response. However, further research needs to be done to confirm our hypothesis. In our study, the overall number of oxPTMs in GFFYTPK_23-29_ was higher after exposure to argon plasma conditions when compared to treatment with helium condition. This can be argued by the dominant role of H_2_O_2_ and nitrogen species (NO, NO_2_^−^, NO_3_^−^), which are more prominent in argon conditions than singlet oxygen (^1^O_2_) and HOCl in helium plasma [56,57]. In line with these findings, we highlight again that the highest number of oxPTMs was found in oxIns III, which has the strongest stimulatory effect on proliferation in various cell lines.

In previous studies, oxidized insulin correlated with altered immunogenicity, e.g., promoted IgG production [58] or increased IgG autoantibody binding to OH-oxidized insulin and associated CD4^+^ and CD8^+^ T cell response [45]. Furthermore, Sharma and colleagues summarized non-oxidative PTMs, such as glycosylation, phosphorylation, acetylation, and SUMOylation, that play important roles in Diabetes due to autoreactivity [59]. In the present study, we did not investigate the immunogenicity of plasma-oxidized insulin, as we previously saw increased activity in antigen-presenting cells after incubation with oxIns I [60]. Still, we detected increased secretion of pro-inflammatory cytokine IL6 in THP-1 monocytes after stimulation with most of the oxidized insulin variants. Furthermore, oxIns V led to increased pro-inflammatory cytokine secretion of TNFα, IL6, and IL8, suggesting to promote a chronic inflammatory condition. However, altered results were obtained when incubation cells with insulin after exposure to other plasma conditions, as we observed reduced TNFα and MCP-1 secretion after incubation with oxINs II and III in TK6 T-cells.

This study aimed to evaluate the biological consequences of insulin oxidation in the context of hyperglycemia. To address this, oxidized insulin variants were artificially generated based on gas plasma-generated ROS in a controlled environment and not as a consequence of ROS release by cells under hyperglycemic conditions. However, the chosen cell culture conditions may have biological consequences far beyond the functional alterations of the oxidized insulin variant that were not addressed in the present study. In biological systems, mitochondria are a major source of ROS generation, and mitochondrial dysfunction has been linked to excess free radical generation during hyperglycemia early on [61]. Intriguingly, the evaluation of mitochondrial density in patients with type 2 diabetes showed a lower number of mitochondria compared to those of age-matched insulin-sensitive individuals [62]. Likewise, NMR analysis on individuals with insulin resistance revealed a marked reduction of mitochondrial oxidative and phosphorylation activity in their muscle and liver tissues, suggesting a direct association between mitochondrial density and insulin resistance under hyperglycemic conditions [63]. It is evident that mitochondrial ROS serve as a second messenger and enhance insulin sensitivity, e.g., via oxidative modification of the insulin receptor [64]. In this view, a persistent oxidative challenge under hyperglycemic conditions is suggested to induce systemic adaptions that improve both insulin action and antioxidant capacity [65]. On the other hand, it is known that hyperglycemia-induced ROS promote insulin resistance in rodent animal models and humans [66]. Thus, it would be interesting to identify the threshold that determines the switch of ROS from an enhancer to a suppressor of insulin sensitivity.

Besides alterations in the cellular response of oxidized insulin, our study provides evidence for affected blood sugar homeostasis. Several therapeutics deal with the problem of oxPTMs, which arise during the drug manufacturing process or later and affect the structure or function of the biomolecules [67,68]. For instance, loss of antigen-binding, aggregation, or reduced stability was shown in therapeutic antibodies [69] or reduced protein activity, such as interferon [70] or erythropoietin [71]. Attempting to identify critical oxidative modifications and options to modify gas plasmas specifically to enrich such modifications, we correlated optical emission profiles with oxidative modifications (Fig. S7). This revealed emission bands of 357 nm (nitrogen) and 763 nm and 772 nm (argon) to strongly correlate with, e.g., hydroxy and quinone groups. In the example of A549 responses, for instance, there was a clear association with gas plasma-generated 357 nm emission line intensity, insulin nitration (Fig. S7), and glucose uptake in A549 as well as INS1R and GLUT expression in the other cell lines tested (Fig. S8). Investigating such links may reveal more distinct functions of specific peptide and protein modifications in human physiology and pathology.

## 4 Conclusion

Chronic hyperglycemia is a potent inducer of free radical production, leading to oxidized insulin variants. Oxidative protein modifications can cause functional changes but evidence of the cellular effects of oxidized insulin variants is lacking. Using gas plasma technology to create a modular ROS environment, this study identified a stimulatory effect of oxidized insulin variants on proliferation and metabolism in various cell lines. Exposure to oxIns further affected blood sugar homeostasis *in ovo*. Evaluation of oxPTMs after exposure to different plasmas via nano LC-MS identified a high number of oxPTMs in the B chain sequence. Modifications B chain residues relevant to the direct interaction of insulin with its receptor suspected an altered binding, and might explain altered cellular responses. Moreover, correlation analysis linked nitrogen plasma emission lines and insulin nitration to elevated glucose uptake. Taken together, our results underline the importance of evaluating the effects of protein oxidation on health and disease.

## 5 Experimental section

### 5.1 Cell culture

All cells were cultured in an incubator at a temperature of 37 °C and a CO2 saturation of 5 % and split two to three times per week. Cells were cultured in flasks (Sarstedt, Nümbrecht, Germany) in DMEM medium or RPMI (both Pan-Biotech GmbH, Aidenbach, Germany) with Hams F12K (Thermo Fisher Scientific, Dreieich, Germany) as described in Table 1. For subculturing adherent cells, the medium was removed, and cells were washed with PBS and incubated with Accutase (Pan-Biotech GmbH, Aidenbach, Germany) for 10–15 min to detach. The cell suspension was transferred in a tube and centrifuged at 500×*g* for 5 min before being resuspended in PBS for washing and centrifuged again. The cells were then resuspended in fresh medium, and 10–30 % of the cells were further cultured in a new cell culture flask. The suspension cells (THP-1 and TK-6) were transferred to a tube and centrifuged before being resuspended in fresh medium and transferred to a new cell culture flask. The cell lines used in this study were selected in a pilot screening of eighteen different cell lines based on INS1R and GLUT4 expression. For further evaluation, cell lines representing high, average, and low expression levels of the respective receptors were selected.

**Table 1:** Cell lines and cell culture media.

Cell line	medium
A549	DMEM (4.5 g/L Glucose, 10 % FCS, 1 % l-Glutamine, 1 % Penicillin/Streptomycin
CHO-INSR-1284	Ham's F12K (10 % FCS, 1 % l-Glutamine, 0,68 % Hygromycin)
HepG2	DMEM (1.0 g/L Glucose, 10 % FCS, 1 % l-Glutamine, 1 % Penicillin/Streptomycin)
INS1E	RPMI (10 % FCS, 1 % l-Glutamine, 1 % Penicillin/Streptomycin, 1 % sodium pyruvate, 1 % HEPES)
MCF-7	DMEM (4.5 g/L Glucose, 10 % FCS, 1 % l-Glutamine, 1 % Penicillin/Streptomycin)
THP-1	RPMI 1640 (10 % FCS, 1 % l-Glutamine, 1 % Penicillin/Streptomycin)
TK6	RPMI 1640 (10 % FCS, 1 % l-Glutamine, 1 % Penicillin/Streptomycin)

### 5.2 Cell experiments

For experiments, cells were harvested in their exponential growth phase at 70 % confluence and counted via flow cytometry (Cytoflex S instrument, Beckman-Coulter, Krefeld, Germany) using 4′,6-diamidine-2-phenylindole (DAPI; Carl Roth, Karlsruhe, Germany) (1 μM) for live-dead discrimination. Depending on the cell line, a different number of cells per well was seeded in a 96-well plate in 100 μl medium. Two-thousand five-hundred fast-growing A549 and 5000 MCF-7 and HepG2 cells were seeded per well in DMEM high glucose (4.5 g/l). THP-1 cells were seeded at 20000, TK6 cells at 10,000, and INS1E at 30,000 cells per well in RPMI. For experiments with CHO cells, 5000 cells were seeded per well in Hams-F12K medium. Experiments testing different concentrations of native insulin and the effects of oxIns were performed as follows: At the 0h time point, the cells received increasing concentrations of insulin (final concentrations (fcc) were 0.01, 0.1, 1, 10, and 100 μg/ml in PBS), the control group received insulin-free PBS. For the experiments with oxIns, the cells were incubated with the different gas conditions of oxIns (10 μg/ml in PBS), while the control received gas-treated insulin (10 μg/ml in PBS). All cell culture experiments were carried out in three to six biological replicates, each with three technical replicates unless otherwise stated.

### 5.3 Metabolic activity

Alamar blue (Biomol, Hamburg, Germany) assay was used to investigate metabolism. The active ingredient resazurin is blue and permeable to the cell wall and was given to the cells 3h prior to detection time at a final concentration of 100 μM. Resorufin, a red substance, which is the product of converted resazurin during metabolism, was measured using fluorescence microplate readers (Tecan, Männedorf, Switzerland) at *λ*_ex_ 560 nm and *λ*_em_ 590 nm.

### 5.4 Proliferation assay

Cell number of the A549, HepG2, and MCF-7 cells was determined 72h after cells were incubated with PBS/Ins/oxIns to conclude proliferation. After incubation, supernatants were removed, and cells were washed with PBS and fixed with 4 % paraformaldehyde (PFA; Carl Roth, Karlsruhe, Germany) in PBS for 20 min at room temperature. After removing PFA, the cells were washed again before incubation with 0.5 % Triton X (Thermo Fisher Scientific, Dreieich, Germany) for 10 min at room temperature. The cells were then washed and stained with DAPI (1 μM in PBS). The fluorescence signal from the DAPI staining was then measured in the Tecan Reader (*λ*_ex_ 360 nm and *λ*_em_ 465 nm excitation). THP-1 and TK-6 cell proliferation was determined via flow cytometry. Therefore, cells were seeded in 48-well plates with 100,000 cells (THP-1) and 50,000 cells (TK-6) in 500 μl RPMI medium. After 48 h of incubation with PBS/Ins/oxIns, the cells were centrifuged, washed with PBS, and resuspended in PBS with DAPI (1 μM) for live/dead discrimination. The number of viable cells was determined for proliferation.

### 5.5 Cell viability and surface marker expression

Cells were collected in a 96 V-bottom plate (Nunc; Thermo Fisher Scientific, Bremen, Germany) and washed three times with PBS. Surface marker expression was investigated by incubating the cells with fluorochrome-conjugated antibodies INSR Phycoerythrin (PE) (BioLegend, Amsterdam, The Netherlands) and GLUT4 Alexa Fluor 488 (AF488)) (R&D Systems, Minneapolis, USA). After incubation for 15 min at room temperature in the dark, cells were washed three times with PBS and reconstituted in PBS containing DAPI (final concentration 1 μM). Flow cytometry experiments were performed using a CytoFLEX S or Cytoflex LX device (both Beckman-Coulter, Krefeld, Germany), and data were analyzed using Kaluza 2.2 (Beckman-Coulter, Krefeld, Germany). For cell line screening.

### 5.6 INSR and GLUT4 screening

19 cell lines were screened for INSR and GLUT4, namely: A549, HepG2, MCF-7, HaCat, SCC-4, SCC-9, T47D, Panc-1, PaTuT, MIAPaCa, FaDu, RT-112, CLS-439, T24, SCaBER, JE6 0.1-TPR, TK6, THP-1 and THP-1-R. Cells from each cell line were harvested at 70 % confluence, washed, and resuspended in PBS with DAPI (1 μM) and counted by FACS. 1.5 million cells were taken in a separate tube, centrifuged, and resuspended in FACS running buffer (Miltenyi Biotec, Bergisch Gladbach, Germany). To eliminate autofluorescent signals, the signal intensities of the unstained cells were subtracted from those of the stained cells. The measurements were carried out using a biological replicate with three technical replicates each.

### 5.7 Glucose uptake

Measurements of glucose uptake after incubation with Ins/oxIns were performed using 2-(N-(7-nitrobenz-2-oxa-1,3-diazol-4-yl)amino)-2-deoxyglucose (2-NBDG; Thermo Fisher Scientific, Dreieich, Germany) and determining fluorescent signal by microscopy (adherent cells) or flow cytometry (suspension cells). All cells were seeded and incubated in the incubator for 24 h prior to washing and adding PBS/Ins/oxIns and 2-NBDG (100 μM) in PBS. After 1h incubation in the cell culture incubator, glucose uptake was stopped by removing the supernatants containing 2-NBDG and washing the cells twice with ice-cold PBS. Glucose uptake in THP-1 and TK-6 cells was detected by flow cytometry. The adherent cell lines were measured using high-content imaging in the Operetta CLS (PerkinElmer, Hamburg, Germany). Brightfield illumination and digital phase contrast (DPC) were used to detect the cells. A blue laser with 435/528 nm wavelength (excitation/emission) was used to excite the 2-NBDG. An air lens with 20x magnification and measured without a confocal lens. Before each measurement, wells without 2-NBDG staining were recorded for each cell line to obtain a background signal, which was subtracted from the signal intensities of the samples. The data were evaluated using the Harmony 4.8 software by measuring the fluorescence intensity of the cell nuclei.

### 5.8 Optical emission spectroscopy

Reactive species profiles of different plasma feed gas admixtures were determined by optical emission spectroscopy (OES). Herein, an optical fiber was placed on the axis below the gas plasma effluent and connected to the spectrometer (AvaSpec-3648-USB2, 0.7 nm of spectral resolution; Avantes, Alsdorf, Germany). The distance between the kINPen nozzle and the fiber holder was 2 cm.

### 5.9 Protein treatment with kINPen plasma jet

Insulin (Merck, Darmstadt, Germany) was reconstituted following the manufacturer's instructions and diluted in PBS (1 mg/mL). Before treatment, insulin was centrifuged at 15,000 g at 4 °C for 5 min to prevent premature aggregation of insulin. 200 μl per well were treated with different plasmas using the atmospheric pressure plasma jet kINPen (neoplas, Greifswald, Germany), which is extensively characterized [38], for 30 s at a distance of 8 mm between the nozzle and the liquid. The treatment was carried out using argon or helium (both Air Liquide, Paris, France; purity 99.999 %) at a flow rate of 1 standard liter per minute (slm) with or without a mixture of 2 % molecular oxygen or nitrogen (both Air Liquide, Paris, France). After treatment, evaporated volume was compensated by adding ddH_2_O, and the samples were aliquoted and stored at −20 °C.

### 5.10 Photon correlation spectroscopy

Spectroscopy measurements of native or gas plasma‐treated Ova (100 μg mL−1) using a ZS90 dynamic light scattering (DLS) device (Malvern Instruments, USA) equipped with a helium‐neon laser light source (632 nm). Proteins (Material RI = 1.45, absorption = 0.001) in PBS similar to water (Dispersant RI = 1.33, viscosity = 0.954) were measured in low‐volume disposable cuvettes (ZEN0040). DLS measurements were done at a set angle of 90° and attenuator at 11. The size was assessed at 22 °C, with an equilibration time of 120 s and cuvette position at 3 mm. Backscatter-angled detection was performed at 173° with a scattering collection angle of 147.7°. Each biological replicate was measured in several replicates with minimal time between repeats. Data analysis was carried out from three independent experiments.

### 5.11 Gel electrophoresis, Coomassie, and silver staining

All reagents, buffers, and devices were supplied by Thermo Fisher Scientific unless otherwise stated. For sample preparation, 5 μg of gas-treated insulin or oxInsulin were mixed with 4x NuPAGE LDS sample buffer and loaded without denaturation on a 10‐well 4–12 % Bis‐Tris Gel. The two outer pockets were each filled with 7 μl SeeBlue Plus 2 standards. Gel electrophoresis was performed in the Mini Gel Tank, filled with 1x MES SDS Running Buffer, connected to the power source. The program in the device ran at 120 V, 60 mA and 30 W for 1.5 h. To visualize the protein bands by Coomassie staining, the gel was removed from the gel tank after gel electrophoresis, transferred to a vessel, and washed with ddH_2_O. It was then completely covered with the Coomassie staining solution (4 % Coomassie Brilliant Blue, 80 % methanol, 20 % acetic acid), heated in a microwave for 10 s, and placed on a laboratory shaker for 10 min. The Coomassie staining solution was then discarded, and the gel was washed several times with a destaining solution (20 % methanol and 10 % acetic acid). A photo was then taken of the gel, and the bands were quantified using the ImageJ program. The experiment was carried out twice. For silver staining, the gel was stained using a staining kit following manufactures instruction (Bio-Rad Laboratories GmbH, Feldkirchen, Germany). A photo of the gel was then taken. The experiment was carried out once.

### 5.12 Mass spectrometry and data analysis

To investigate the influence of plasma treatment on the structure of insulin, the samples were examined using mass spectrometry (MS) coupled with liquid chromatography (LC). For this experiment, 100 ng of gas-treated insulin or oxIns were digested using trypsin (Promega, Walldorf, Germany) at an enzyme-substrate ratio of 1:40 overnight at 37 °C. After digestion, samples were dried and reconstituted in 0.5 % formic acid (FA; Merck, Darmstadt, Germany). A Dionex™ UltiMate™ 3000 RSLC nano system was connected to an Orbitrap Exploris™ 480 mass spectrometer using Nanospray Flex ion sources (both Thermo Scientific, Waltham, MA, USA). The temperature of the columns was 40 °C. The peptides were eluted from the trap column and analyzed on a PepMap C18 reversed-phase analytical column using buffer A (0.1 % acetic acid; Merck, Darmstadt, Germany) and buffer B (95:5 ACN; Th Geyer, Berlin, Germany:0.1 % acetic acid) at a Flow rate of 300 nL/min with an elution gradient of 4–40 % buffer B over 50 min. The mass spectrometer was operated in positive polarity mode with a capillary temperature of 250 °C and a spray voltage of 2 kV. It was operated in data-dependent acquisition mode (Top 15), and the peptides were analyzed with a full scan (350–1200 *m/z*, R = 120,000 at 200 *m/z*) with a target of 5 × 10^3^ ions. This was followed by 15 data-dependent MS/MS scans with higher energy collision dissociation (HCD) at a maximum injection time of 50 ms, an isolation width of 1.0 m/z, and a normalized collision energy (NCE) of 30 %, which were carried out in the Orbitrap (R = 15,000 were detected at 200 *m/z*.

An in‐depth analysis of modifications was performed using Byonic software (Proteinmetrics, USA) as a plug‐in into Proteom Discoverer running against an in‐house designed database for oxidative and post‐translational modifications. A custom modification list was used based on previous experiments [57,72]. The raw data were filtered and modifications were considered valid if a protein modification occurred in ≥66 % replicates of a group. In a separate experiment, the peptide SIINFEKL was used to allow conclusions on the reproducibility of the method of obtaining oxidative modifications from gas plasma exposure. To this end, the peptide was gas plasma treated for 20 s or exposed to argon gas only (plasma = off) for 20 s in double-distilled water. Five different samples were treated on five different days. Injection into the mass spectrometer was done in five technical replicates for each of the five samples (25 data points). Experimentally, sample acquisition was done as described above. Data acquisition was performed in DDA mode. LC−MS/MS raw data were analyzed using Proteome Discoverer (version 2.4.1.55; Thermo Fisher, Dreieich, Germany). MS/MS spectra were extracted from the raw files, and searches were performed with custom FASTA files containing the amino acid sequence of SIINFEKL and common background contaminants. Peptide-spectrum match (PSM) and oxPTM identification and quantification were performed with Byonic (Protein Metrics, Cupertino/CA, USA). Parameters for Byonic were set as follows: peptide mass tolerance = 10 ppm, fragment mass tolerance = 10 ppm, cleavage specificity = no cleavages, and total common modifications = 2. Filtering steps by using cut-off values (byonic score ≥250, logProb ≥3, delta mod score ≥10. PSM) were performed to identify valid hits. Valid hits were sorted in Excel. Oxidative modifications were counted for each group by using the Pivot function.

### 5.13 In ovo experiments

Specific pathogen-free (SPF) fertilized chicken eggs were placed in a specialized breeding incubator equipped with a turning unit in horizontal position. Breeding conditions were 37 °C and 65 % humidity throughout the experimental period. After six days of incubation, the pointed pole of the eggs was punctured using a 20 G cannula to create an air hole. The hole was covered with an air-permeable plaster, and eggs were placed back in the static unit of the incubator, now in vertical position. On day 6, the pointed pole of the egg was punctured. Native or oxidized insulin was applied on the CAM on day 11, and the egg was incubated for 1h, 3h, or 5h. Then, the eggshell was windowed at the punctured side with a diameter of approximately 2 cm. A drop of blood was taken carefully, and blood sugar level was measured.

### 5.14 Cytokine and chemokine quantification

Cytokines were measured using multiplex cytokine analysis assay according to the manufacturer's instructions (LegendPlex; BioLegend, Amsterdam, Netherlands). This multiplex is a bead‐based sandwich immunoassay and was measured using flow cytometry (CytoFLEX S; Beckman‐Coulter, USA). For quantification, data analysis software (Vigene Tech, France) was utilized. A separate standard curve was calculated using fifth‐degree polynomials for each analyte, with attention to the analytes' specific detection limits.

### 5.15 Statistical analysis

Graphing and statistical analysis were carried out using *prism* 9 (GraphPad Software, San Diego, California, USA). Comparison of two groups was made using Mann-Whitney test. Comparison of more than two groups was made using one-way analysis of variances (anova). Comparison of more than two groups across different data sets was made using two-way anova. Levels of signiﬁcance were indicated as follows: α = 0.05 (∗), α = 0.01 (∗∗), α = 0.001 (∗∗∗).

## Funding

This work was funded by the German 10.13039/501100002347Federal Ministry of Education and Research (BMBF), grant numbers 03Z22DN11 (to SB) and 03Z22Di1 (to KDW and SB). The funding source had no role in the design of this study or its execution, analyses, interpretation of the data, or decision to publish results.

## CRediT authorship contribution statement

**Ramona Clemen:** Conceptualization. **Wiebke Dethloff:** Conceptualization. **Julia Berner:** Conceptualization. **Paul Schulan:** Conceptualization. **Alice Martinet:** Conceptualization. **Klaus Dieter Weltmann:** Conceptualization. **Thomas von Woedtke:** Conceptualization. **Tilman Grune:** Conceptualization. **Kristian Wende:** Conceptualization. **Sander Bekeschus:** Conceptualization.

## Declaration of competing interest

The authors declare no conflict of interest.

## Data Availability

Data will be made available on request.
