# Supplementary material for: Insulin oxidation and oxidative modifications alter glucose uptake, cell metabolism, and inflammatory secretion profiles
Source: Redox Biol. 2024 Oct 5;77:103372. doi: 10.1016/j.redox.2024.103372 (PMC11492613; doi:10.1016/j.redox.2024.103372)

## Supplemental Figure S1


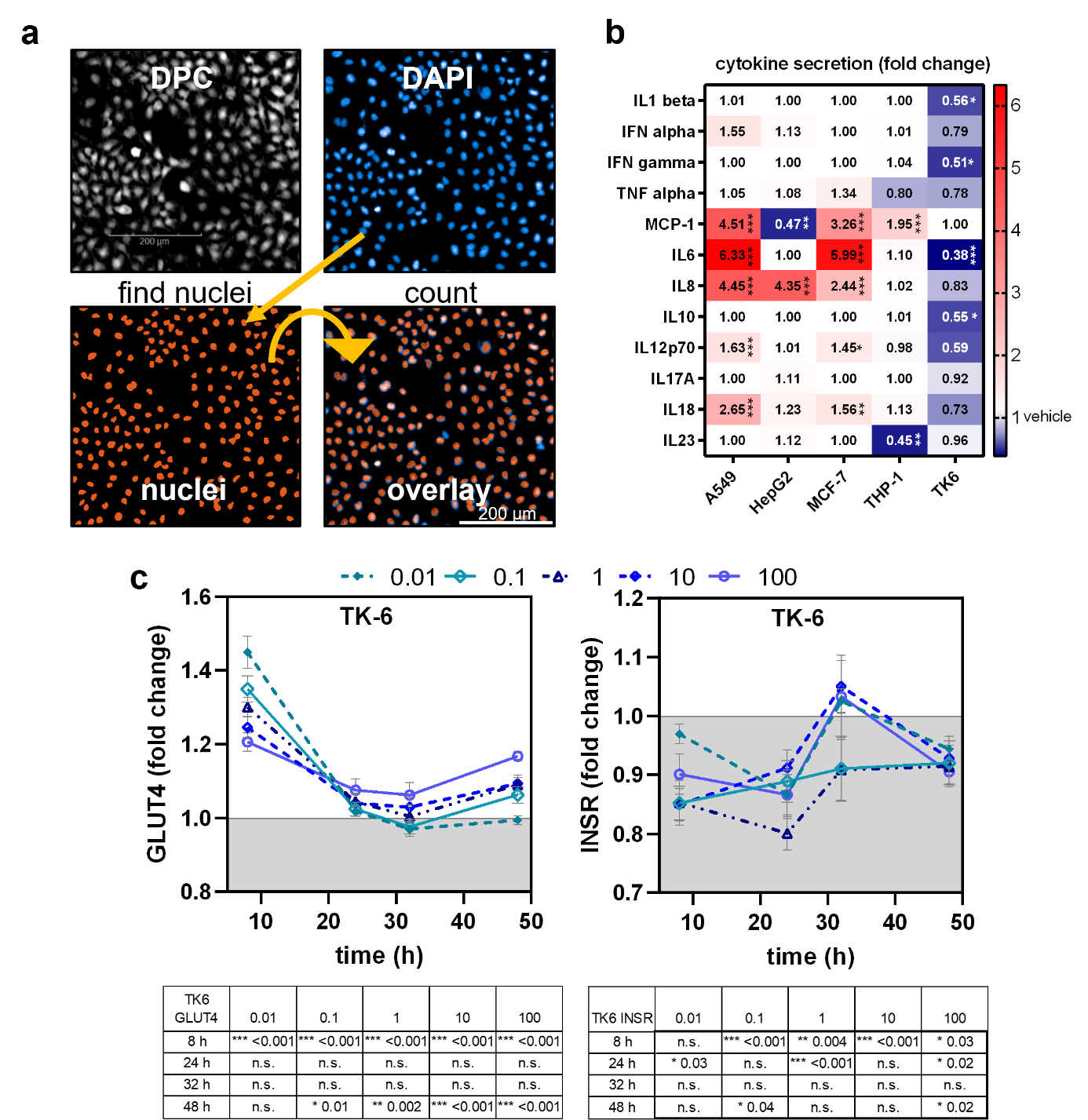


## Supplemental Figure S2


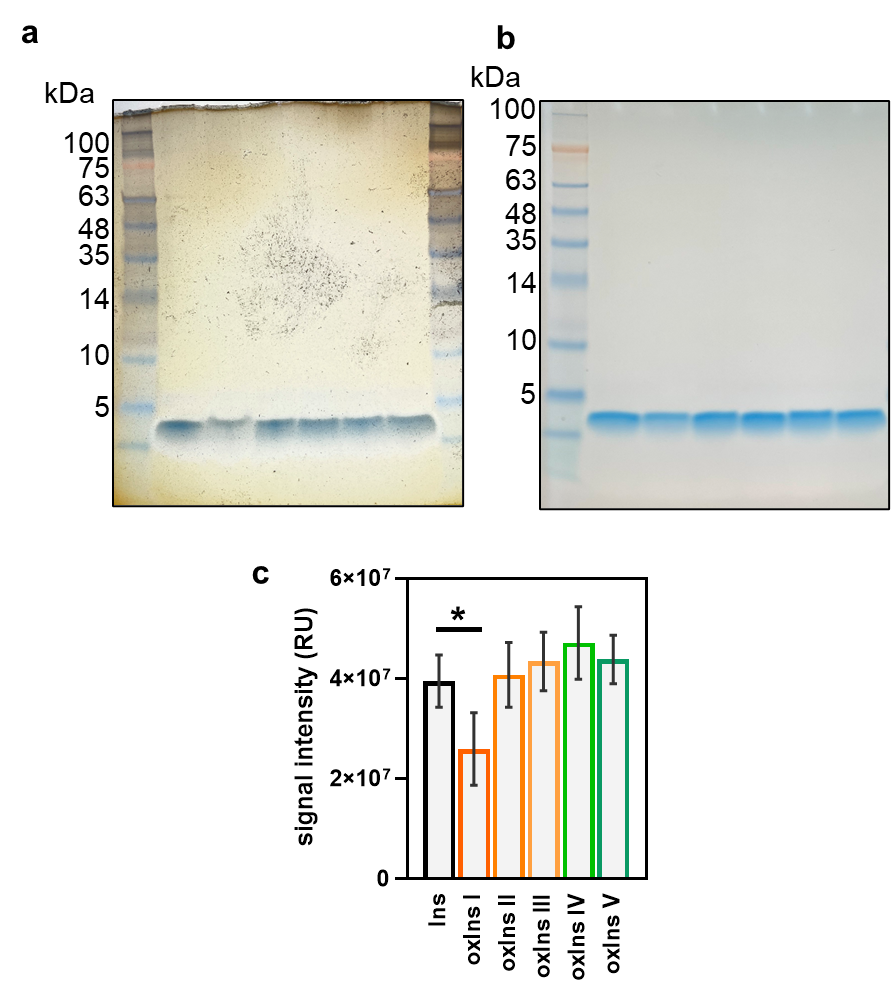


## Supplemental Figure S3


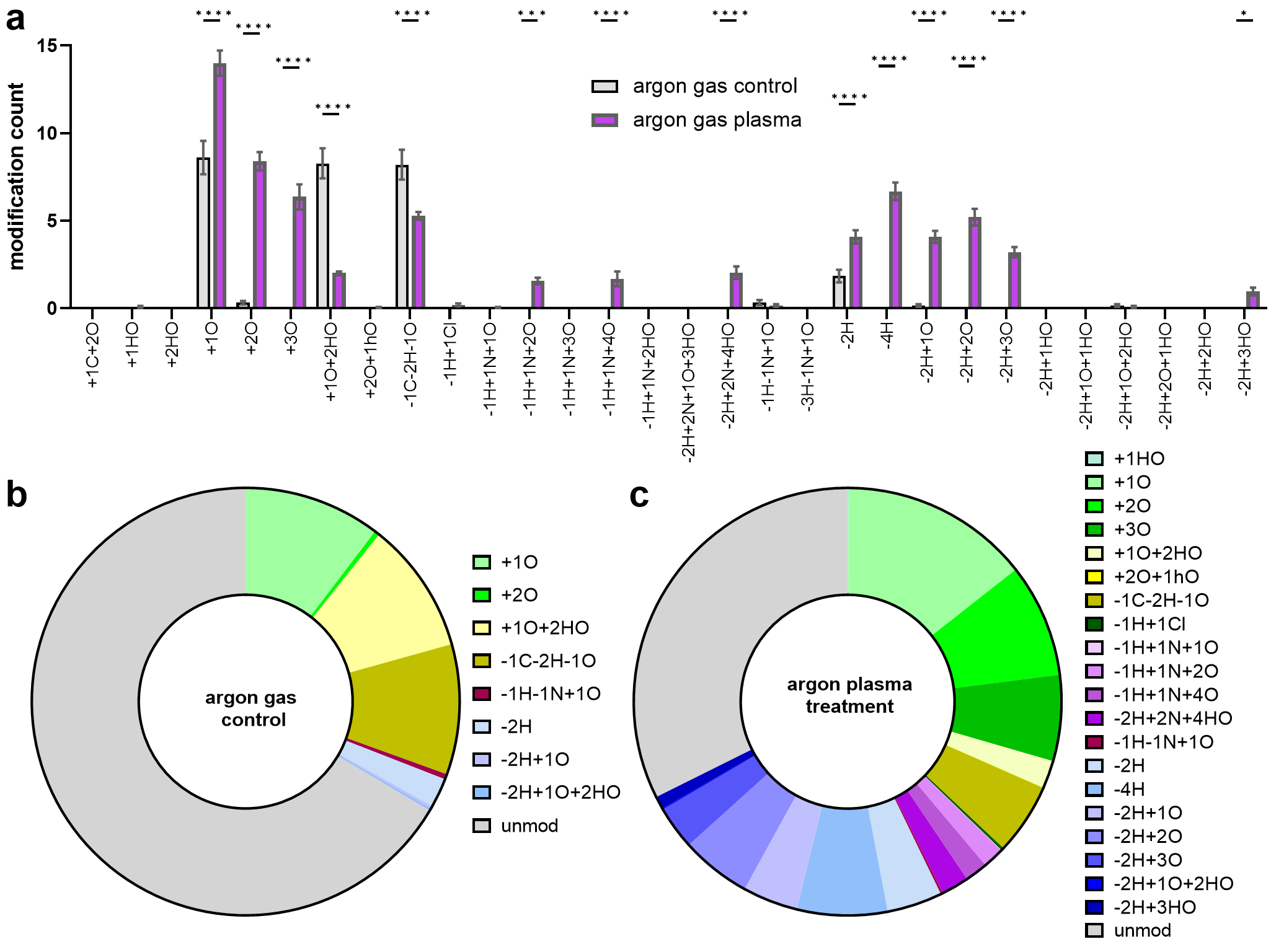


## Supplemental Figure S4


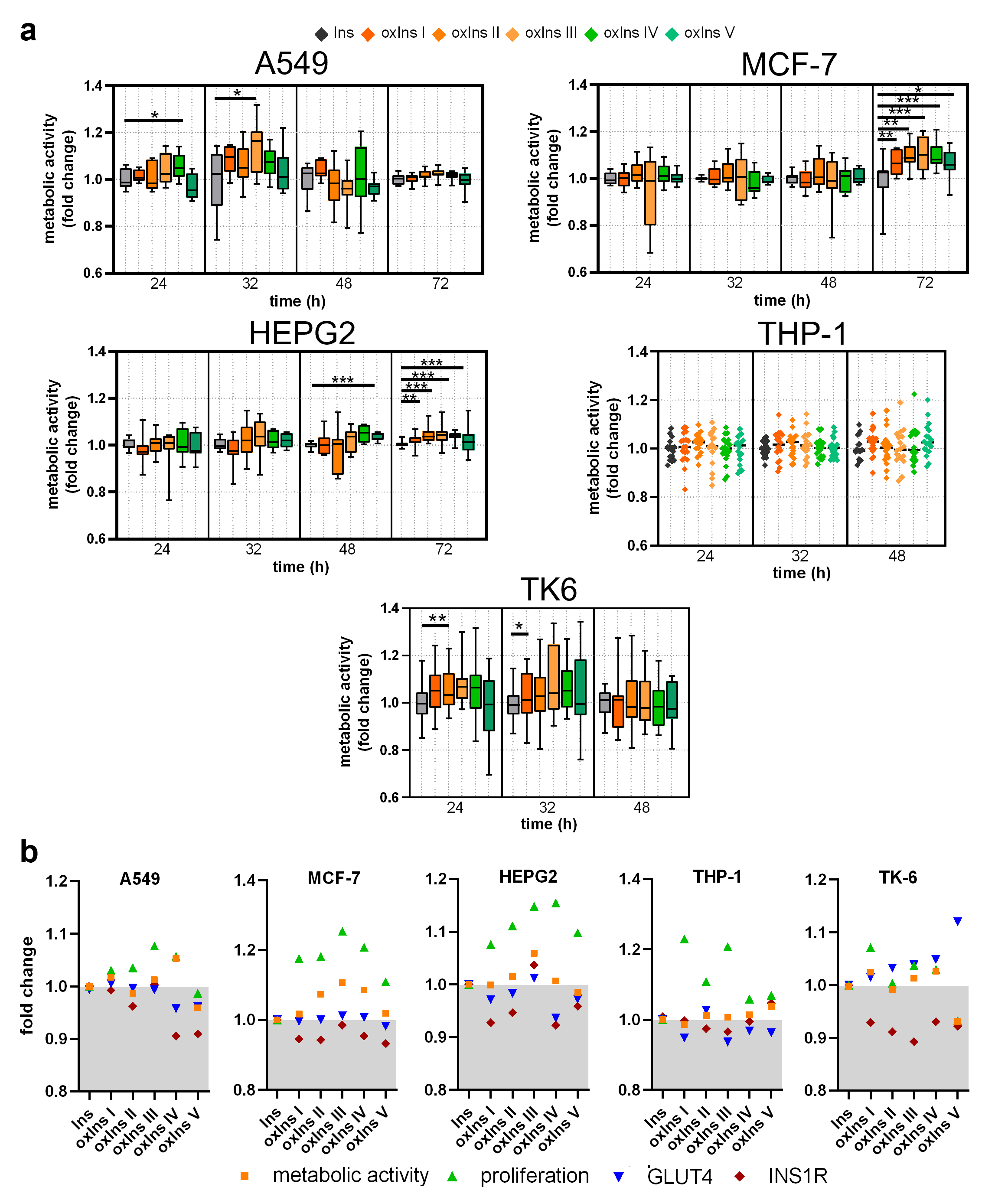


## Supplemental Figure S5


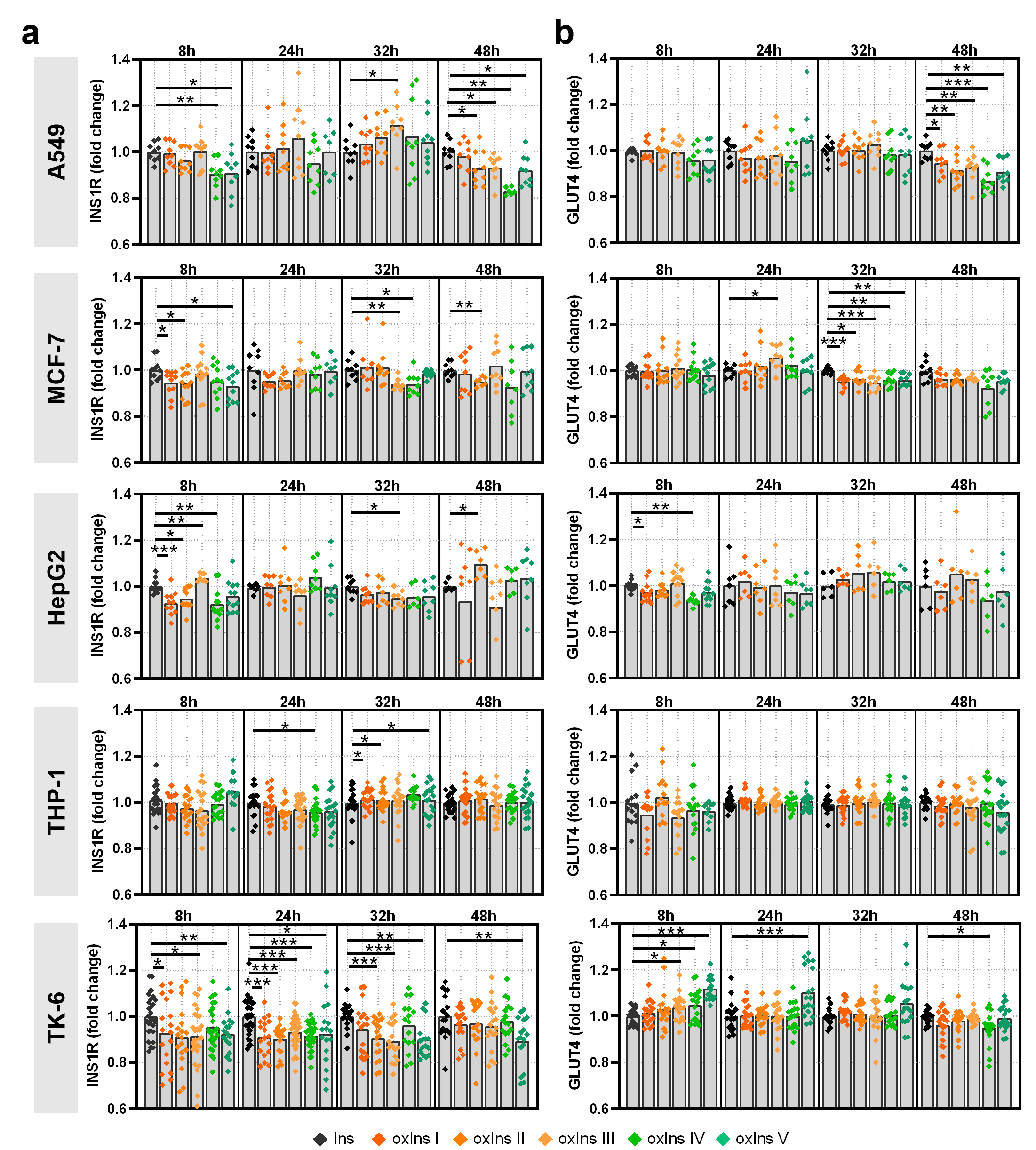


## Supplemental Figure S6


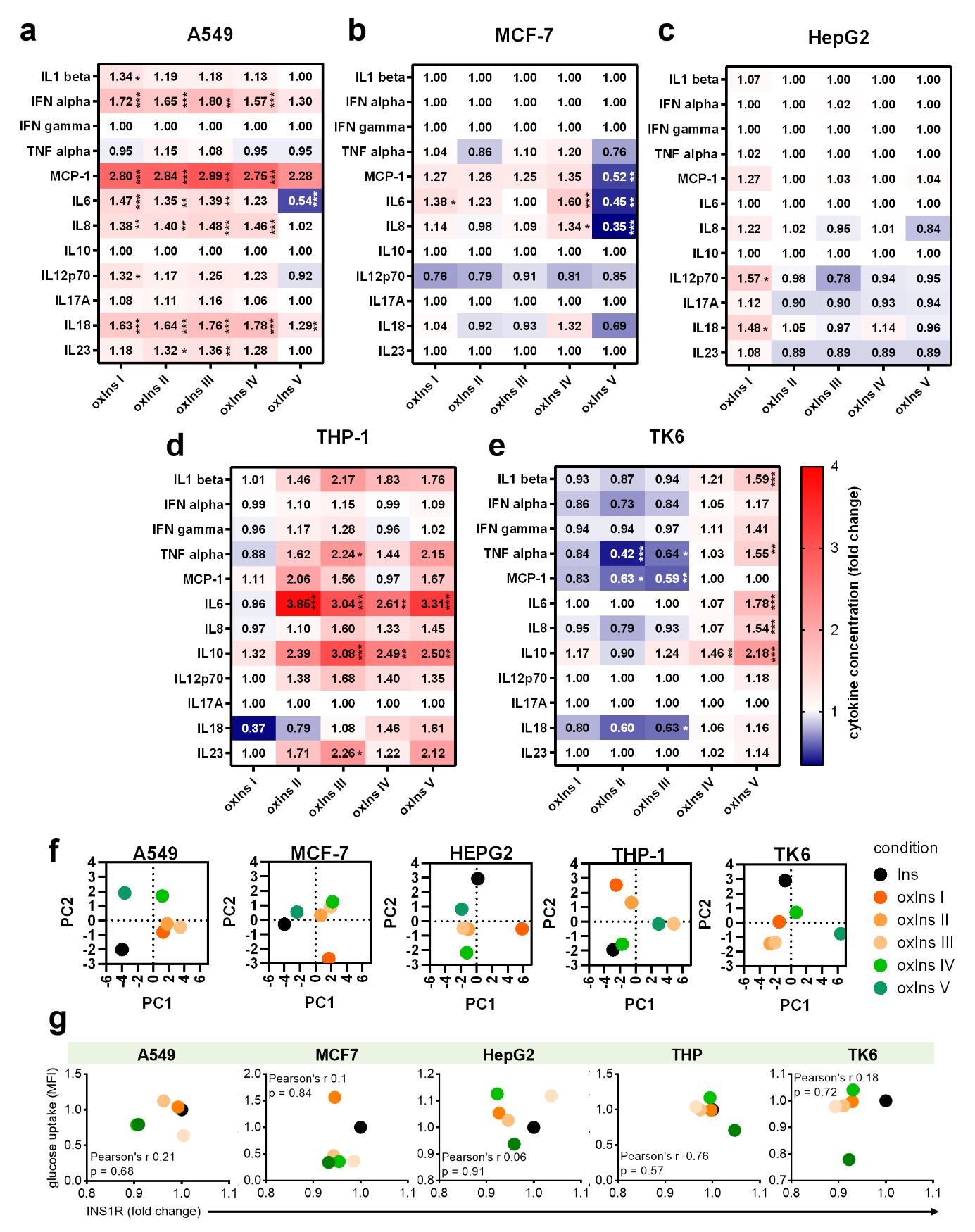


## Supplemental Figure S7

## Supplemental Figure S8


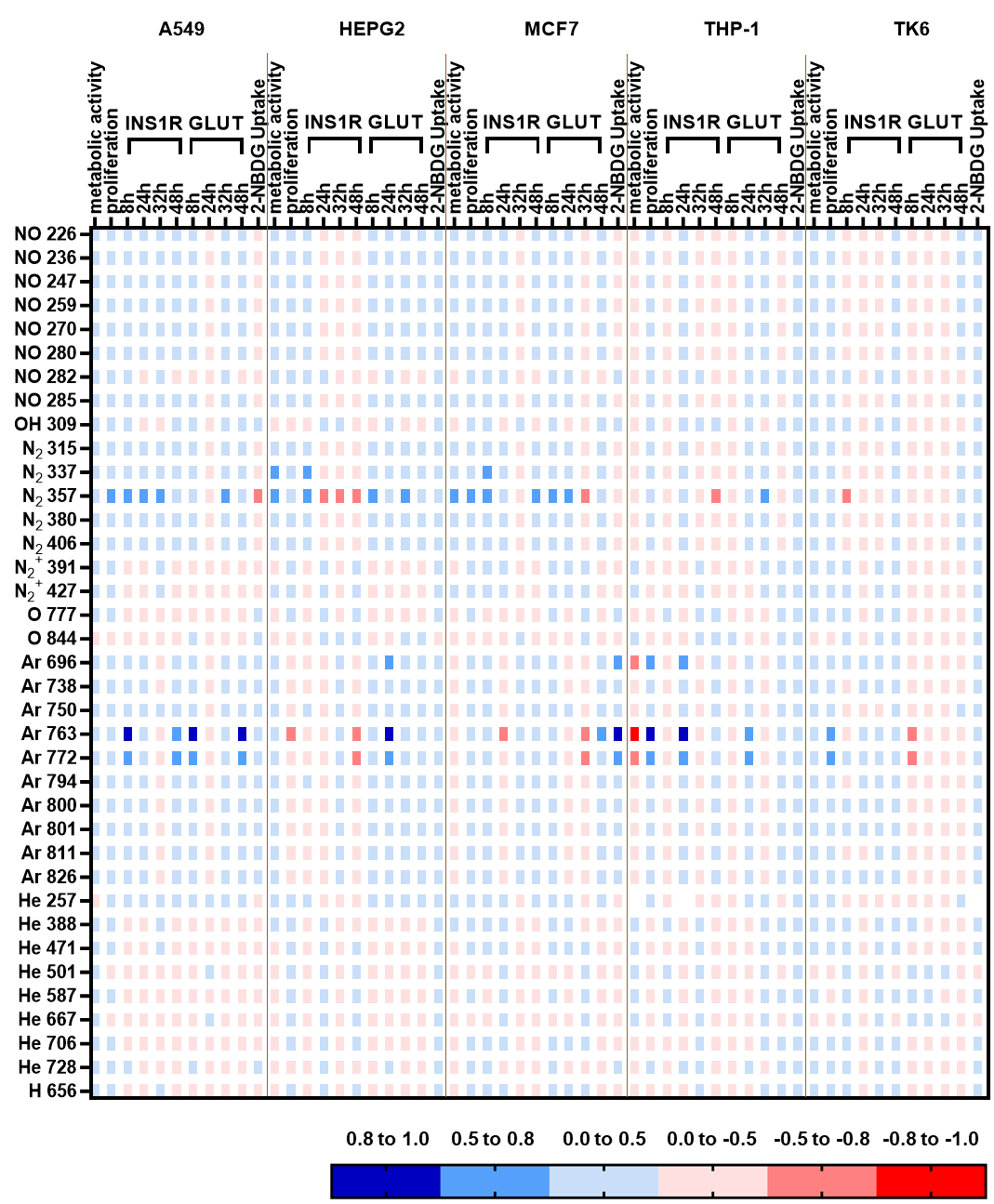

Supplement: Multimedia component 1 [file mmc1.docx]
